# Analyzing the learning curve of vaginal pelvic reconstruction surgery with and without mesh by the cumulative summation test (CUSUM)

**DOI:** 10.1038/s41598-022-11039-5

**Published:** 2022-04-29

**Authors:** Chin-Jui Wu, Kuan-Ju Huang, Wen-Chun Chang, Ying-Xuan Li, Lin-Hung Wei, Bor-Ching Sheu

**Affiliations:** 1 Department of Obstetrics and Gynecology, National Taiwan University Hospital, College of Medicine, National Taiwan University, 15F, No. 8, Chung-Shan South Road, Taipei, 10002 Taiwan; 2 Department of Obstetrics and Gynecology, National Taiwan University Hospital , Hsin-Chu Branch, Hsin-Chu, Taiwan

**Keywords:** Urogenital reproductive disorders, Outcomes research, Ligaments

## Abstract

Women who underwent vaginal pelvic reconstructive surgery with or without mesh consecutively between 2004 and 2018 were retrospectively analyzed to determine the learning curve in vaginal pelvic reconstructive surgery. With cumulative summation (CUSUM) analysis of surgical failure and operation time, we assessed the learning curve of vaginal pelvic reconstructive surgery, including sacrospinous ligament fixation, anterior colporrhaphy, posterior colporrhaphy, and optional vaginal hysterectomy with or without mesh placement. The study is based on two individual surgeons who performed vaginal pelvic reconstructive surgery with or without mesh. Two hundred and sixty-four women with stage III or IV pelvic organ prolapse underwent vaginal pelvic reconstructive surgery by surgeons A or B. The median follow-up time of 44 months ranged from 24 to 120 months. Surgical proficiency was achieved in 32–33 vaginal pelvic reconstructive surgery procedures without mesh and 37–47 procedures in the same surgery with mesh. The total surgical success rates for surgeons A and B were 82.2% and 94.1%, with median follow-up times of 60 and 33 months, respectively. More procedures were needed for the learning curve of vaginal pelvic reconstructive surgery with mesh. Having crossed the proficiency boundary, the surgical success rate and operation time were improved.

## Introduction

Pelvic organ prolapse (POP) is a complex entity that comprises the anterior, posterior, and apical compartments, each requiring a separate stage of surgical treatment. Pelvic reconstructive surgery is divided into the repair of the different compartments, with or without mesh. Studies that address the outcomes and complications of pelvic floor reconstructive surgery usually state that the surgical procedure was "performed by an experienced surgeon," yet little data are available to clarify how many surgeries are needed for surgical skill to be considered sophisticated enough to merit this designation. The American Urogynecology Society advocates that surgeons performing intricate pelvic floor reconstructive surgery should have adequate experience and training to manage the inherent complex anatomy and complications^1^.

The cumulative summation (CUSUM) test was initially designed for industrial quality control and later adopted to monitor the learning curve in medicine^2,3^. The CUSUM test sequentially analyzes changes in the process under scrutiny, determining after each procedure whether the process is "in control" (performing at an acceptable level) or "out of control" (performing at an unacceptable level). The CUSUM curve can readily show the trends and outcomes of consecutive events in the figure. When applied to the concept of a learning curve in surgery, the CUSUM curve can be used to determine when a proficient surgeon has crossed the ordinary level and entered into a more stable one^4^. Currently, a wide variety of procedures and operations in gynecology, from embryo transfer to robotic sacrocolpopexy, use CUSUM to analyze the proficiency of doctors^5,6^.

Pelvic reconstructive surgeries can be divided into vaginal and abdominal approaches. Two studies calculated the learning curve of laparoscopic and robotic pelvic reconstructive procedures^6,7^. Very little data regarding vaginal procedures are available. The literature contains more studies of vaginal pelvic reconstructive surgeries, but those focusing on surgical proficiency of the vaginal approach are lacking^8,9^. In addition, proficiency becomes more critical with the application of transvaginal mesh. The use of mesh reinforces pelvic structures and native tissue repairs but also raises issues of mesh-related complications^10,11^. One study analyzed 10,000 transvaginal mesh surgeries and concluded that the surgeon's experience and technique are critical to enhancing the success rate and reducing complications^12^.

The objective of this study was to examine the learning curve of vaginal pelvic reconstructive surgery, including sacrospinous ligament fixation and concomitant anterior and posterior colporrhaphy with or without mesh. The study could help build evidence-based training programs and predict obstacles they may encounter.

## Results

### Demographic data

A total of 264 patients who underwent pelvic reconstruction surgery were analyzed. Among them, 162 patients received pelvic reconstruction without mesh by surgeon A, and 102 patients received pelvic reconstruction with mesh by surgeon B (Table 1). Surgeon A's patients were 65.6 ± 10.5 (mean ± SD) younger than surgeon B's patients (68.4 ± 11.2). Because of the surgeon's preference, patients who received pelvic reconstruction without mesh were significantly more likely to have concomitant and previous hysterectomy than those who received surgery with mesh (p < 0.05). Other parameters, including BMI, parity, menopausal status, hypertension, diabetes mellitus, and POP-Q stage, did not differ significantly.

**Table 1:** Baseline characteristics of patients for the two surgical approaches by individual surgeon.

Variable	Total (n = 264)	Pelvic reconstruction without mesh (n = 162)	Pelvic reconstruction with mesh (n = 102)	p-value
Age, year	66.7 ± 10.8	65.6 ± 10.5	68.4 ± 11.2	0.022*
BMI, kg/m^2^	24.5 ± 3.32	24.7 ± 3.16	24.2 ± 3.54	0.201
Parity, median(range)	3 (0–9)	3 (0–9)	3 (0–9)	0.760
Menopause,n	194 (73.5)	116 (71.6)	78 (77.2)	0.627
Hypertension, n	84 (31.8)	51(31.5)	33(32.3)	0.991
Diabetes	40(15.2)	19(11.7)	21(20.6)	0.098
Previous hysterectomy	64(24.2)	40(24.7)	8(7.8)	< 0.001*
Concomitant hysterectomy	117(44.3)	106(86.9)	3(3.2)	< 0.001*
**POP-Q stage**				
3	199(75.3)	122(75.3)	77(75.5)	0.998
4	65(24.6)	40(24.7)	25(24.5)	0.997

Data are presented as the mean ± standard deviation, median with range, or number (%); Pearson's chi-squared test, independent sample t-test, and Mann–Whitney test were used for categorical data, mean values, and median values, respectively, unless otherwise specified.

*BMI* body mass index, *POP-Q* pelvic organ prolapse quantification.

*Indicates significant p value < 0.05.

### CUSUM analysis of surgical failure rate

**Figure 1:**
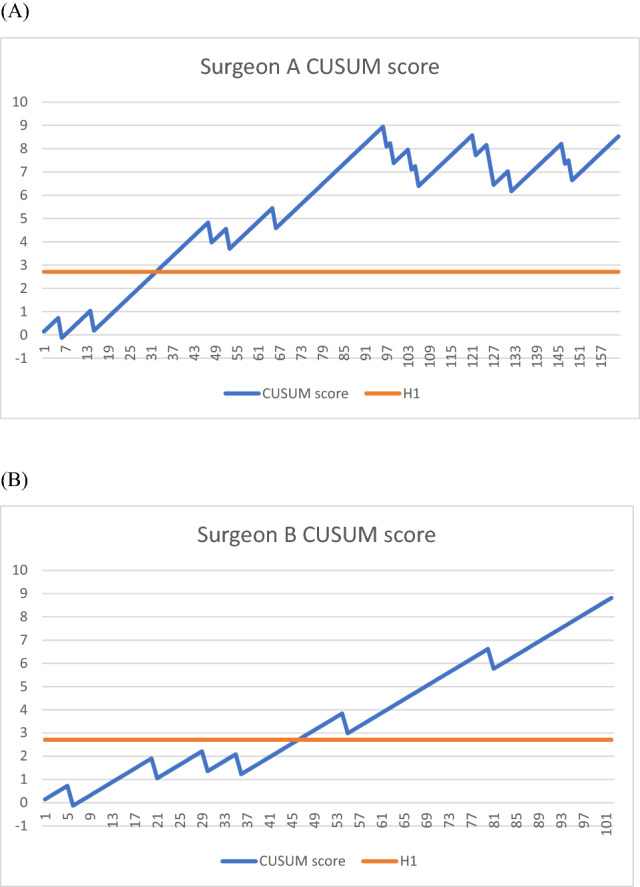
Cumulative sum control chart analysis of surgical failure. The learning curve of vaginal pelvic reconstruction surgeries. The x-axis indicates the number of procedures performed. The y-axis indicates the cumulative sum of success and failure of the surgical team in terms of surgical failure. Surgical failure is defined as recurrent prolapse beyond the hymen in 24 months postoperatively. The H1 line (orange) is designed to detect surgical proficiency. Proficiency is obtained when the graph crosses H1(2.709). Cumulative sum control chart analysis is based on an acceptable failure rate of 10% and an unacceptable failure rate of 20%. **(A)** Vaginal pelvic reconstruction without mesh performed by surgeon A. **(B)** Vaginal pelvic reconstruction with mesh performed by surgeon B.

Figure 1 shows the CUSUM analysis of surgical failure. We calculated two cohorts: surgeon A and surgeon B. Vaginal reconstructive surgery without mesh (SSLF, anterior and posterior colporrhaphy, optional hysterectomy) was the operation method of surgeon A. Vaginal reconstructive surgery with mesh was the operation method of surgeon B. Surgical failure was frequently encountered in the early cases of the series in both cohorts. In case of a failure, the graph falls by 0.855. In the chance of success, the graph rises by 0.145. The unacceptable recurrence threshold, the H1 line, is presented as a horizontal line at 2.709. A constant rate of success above the breakthrough H1 line indicates that proficiency was achieved. Surgical proficiency according to surgical success was defined as no recurrence in postoperative 24 months. The surgical proficiency was stabilized after 33 cases in surgeon A's cohort and 47 cases in surgeon B's. The overall success rates for surgeons A and B were 82.2% and 94.1%, respectively, with median follow-up times of 60 and 33 months.

### CUSUM analysis of operation time

**Figure 2:**
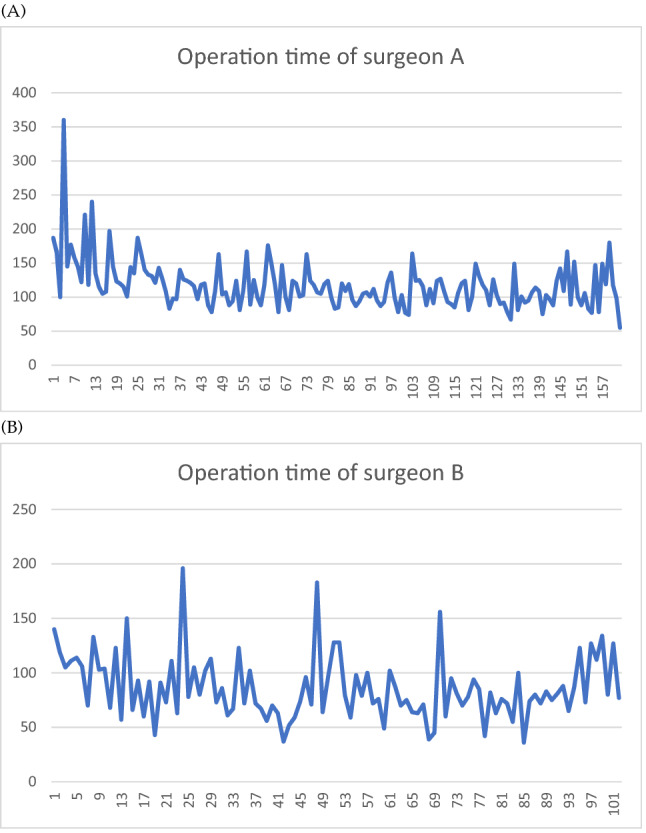
The operation time of pelvic reconstruction without or with mesh. Operation time were recorded. The x-axis indicates the number of procedures performed. The y-axis shows surgery time (minutes). **(A)** Vaginal pelvic reconstruction without mesh performed by surgeon A. **(B)** Vaginal pelvic reconstruction with mesh performed by surgeon B.

**Figure 3:**
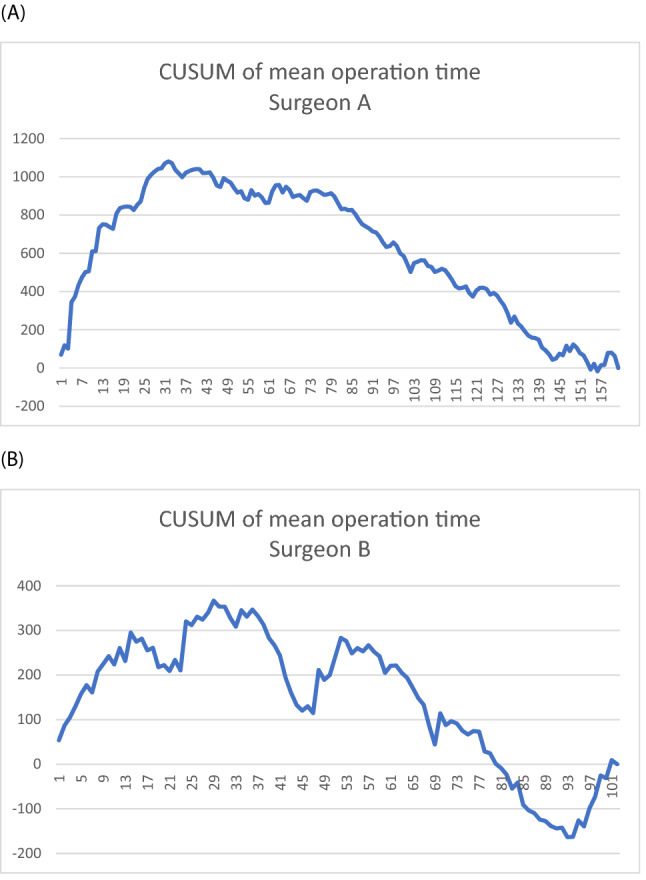
CUSUM of mean operation time. The x-axis indicates the number of procedures performed. The y-axis indicates the cumulative surgery time (minutes) compared to the mean surgery time. When the performance time is longer or shorter than the mean surgery time, the graph rises or falls with the absolute difference in minutes. Because the rising or falling of the graph is based on the mean surgery time, the graph ends at 0 min. **(A)** Vaginal pelvic reconstruction without mesh performed by surgeon A; operation time dropped after 32 procedures. **(B)** Vaginal pelvic reconstruction with mesh performed by surgeon B; operation time dropped after 37 procedures.

The operation time of surgeon A's cohort was 117 ± 35.3 min (mean ± SD), and that of surgeon B's cohort was 86.3 ± 29.4 min. The mean operation time of surgeon A declined from an average of 154 min for the first 25 cases to 110 min for the last 25 cases (Fig. 2A). Surgeon B had a lower initial average of 98 min for the first 25 cases and dropped to 83 min for the last 25 cases (Fig. 2B). The lower concomitant vaginal hysterectomy rate may explain the lower mean operation time of surgeon B than surgeon A. To calculate proficiency in operation time by CUSUM analysis, we found that the peak operation time of surgeon A was case No. 32 (Fig. 3A), and that of surgeon B was case No. 37 (Fig. 3B). Corresponding to the CUSUM analysis of the surgical success rate, pelvic reconstruction with mesh requires a greater number of operations to achieve a stable surgical condition. There are more turning points in the graph of surgeon B (Fig. 3B) compared with surgeon A. These turning points reflect minor modifications made during the pelvic reconstructive surgery when it took more time to tailor and adjust the mesh position.

### Characteristics of the surgeon A and B cohorts per 25-surgery tier

Table 2 shows the baseline characteristics per 25 surgeries, forming seven subgroups in surgeon A's group and four in surgeon B's group. The intergroup comparison showed no significant difference in age, BMI, parity, menopausal status, underlying hypertension, diabetes mellitus, complications, or POP-Q stage. The previous hysterectomy rates were significantly different among surgeon A's cohort subgroups. The previous hysterectomy rates may have influenced the concomitant hysterectomy rates and may have negatively impacted the results of the CUSUM analysis of operation time.

**Table 2:** Characteristics and procedures per 25 surgeries of surgeons A and B.

Variables	Age, years	Body mass index, kg/m^2^	Parity, n (range)	Postmenopausal, n (%)	Hypertension, n (%)	Diabetes mellitus, n (%)	Previous hysterectomy, n (%)	Concomitant VTH, n (%)	POPQ stage III, n (%)	POPQ stage IV, n (%)	Complication, n (%)
**Surgeon A**											
0–25	64.28 ± 13.39	26.29 ± 3.42	4 (2–7)	18 (72)	8 (32)	3 (12)	9 (36)	13 (52)	20 (80)	5 (20)	1 (4)
26–50	65.04 ± 7.90	25.62 ± 3.84	3 (1–6)	18 (72)	7 (28)	4 (16)	10 (40)	11 (44)	18 (72)	7 (28)	2 (8)
51–75	68.88 ± 8.79	24.90 ± 2.83	3 (1–9)	19 (76)	8 (32)	1 (4)	5 (20)	19 (76)	19 (76)	6 (24)	3 (12)
76–100	63.40 ± 9.82	24.46 ± 2.75	3 (0–6)	14 (56)	6 (24)	3 (12)	1 (4)	21 (84)	19 (76)	6 (24)	3 (12)
101–125	67.00 ± 11.36	24.41 ± 3.21	3 (1–8)	16 (64)	10 (40)	4 (16)	5 (20)	20 (80)	17 (68)	8 (32)	3 (12)
126–150	64.16 ± 10.31	24.39 ± 3.20	3 (0–7)	21 (84)	9 (36)	3 (12)	3 (12)	18 (72)	21 (84)	4 (16)	1 (4)
151-end (n = 12)	67.58 ± 11.96	23.34 ± 2.51	2.5 (1–6)	10 (83)	6 (50)	1 (8)	7 (58)	4 (33)	8 (67)	4 (33)	0 (0)
p value	0.507	0.263	0.036	0.355	0.755	0.873	0.002	0.002	0.849	0.849	0.692
**Surgeon B**											
0–25	67.40 ± 13.30	23.56 ± 2.74	3 (1–7)	22 (88)	9 (36)	6 (24)	5 (20)	1 (4)	15 (60)	10 (40)	6 (24)
26–50	70.68 ± 8.46	23.75 ± 2.99	3 (2–8)	23 (92)	10 (40)	8 (32)	0 (0)	0 (0)	22 (88)	3 (12)	5 (20)
51–75	68.00 ± 8.12	25.74 ± 3.81	3 (0–6)	23 (92)	10 (40)	8 (32)	0 (0)	1 (4)	17 (68)	8 (32)	8 (32)
76-end (n = 27)	66.56 ± 13.73	24.33 ± 4.10	3 (1–9)	22 (88)	9 (36)	3 (12)	1 (3.8)	1 (3.8)	22 (88)	4 (16)	9 (36)
p value	0.719	0.112	0.5466	0.974	0.938	0.354	0.03	0.799	0.099	0.099	0.521

Data are given as the mean ± standard deviation, median (range) and n (%); one-way analysis of variance, the Kruskal–Wallis test, Pearson's Chi-squared test, and Fisher's exact test were used to compare mean, median, and nominal variables between groups; post hoc tests were used in case of significance.

**Figure 4:**
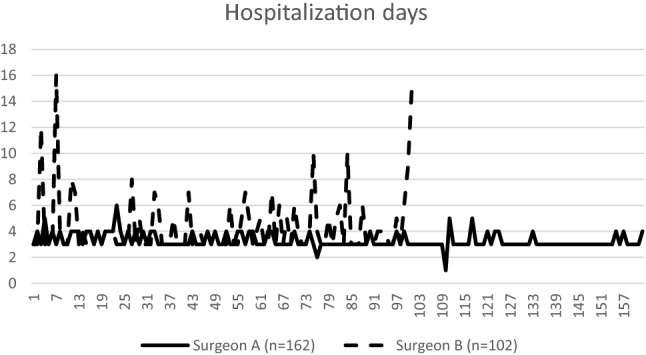
The postoperative hospitalization days. The x-axis indicates the number of procedures performed. The y-axis indicates the postoperative hospitalization time (days). The solid line shows surgeon A's series: vaginal pelvic reconstruction without mesh. The dotted line shows surgeon B's series: vaginal pelvic reconstruction with mesh. The solid line ends at procedure No. 162, and the dotted line ends at procedure No. 102.

The total complication rates of surgeon A and surgeon B were 6% and 27%, respectively. The majority, 20.5%, of the complications in surgeon B's group were postoperative higher residual urine, defined as more than 150 ml after voiding three times. The postoperative days of hospitalization are shown in Fig. 4. The mean postoperative days of surgeon A's group were 3.28 ± 0.57 days, and that of surgeon B's group was 4.48 ± 2.62 days. Nine patients who presented more than seven postoperative days were recorded in surgeon B’s group (Fig. 4). Six of them were because of high postvoid residual urine. Two were because of their medical diseases: pneumonia or thrombocytopenia. One was a urinary tract infection. The high postvoid residual urine was regarded as the short-term complications of mesh and maybe because the mesh was placed extending to the proximal urethra. It had improved along days and did not require a re-operation.

Combining the results of the CUSUM analysis of surgical success and operation time, surgical proficiency can be achieved in 32–33 cases of pelvic reconstructive surgery without mesh. Pelvic reconstructive surgery with mesh was steady after 37–47 patients.

## Discussion

Here, we presented the learning curve of vaginal pelvic reconstructive surgeries with or without mesh by two surgeons. The total success rates for surgeons A and B were 82.2% and 94.1%, respectively. The operation time was 117 ± 35.3 min for surgeon A and 86.3 ± 29.4 min for surgeon B. This result is comparable to most previous studies^13,14^. Our result may be worse because we included procedures the surgeon may not have become adequately proficient at performing. Vaginal pelvic reconstructive surgery may consist of one or more surgeries. In our study, we chose SSLF, anterior colporrhaphy without mesh, and posterior colporrhaphy with or without mesh. These operation methods are most often performed concomitantly. For optional vaginal hysterectomy, surgeon A performed vaginal hysterectomy for 86.9% of the patients with an intact uterus. Surgeon B was prone to preserve the uterus and performed a hysterectomy on only 3.2% of the patients. Although hysterectomy adds surgical time, cost, and morbidity, a recent meta-analysis showed that hysterectomy does not significantly affect surgical outcome^8,15–17^.

Surgical success is the expectation of both the surgeon and patient. Pelvic reconstructive surgery tends to fail over time^18,19^. Our study analyzed three parameters: surgical recurrence, operation time, and hospital stay. We found that hospital stay was not a suitable parameter because the medical and insurance system highly influences its length. Patients with high postoperative residual urine may be discharged with a Foley catheter or hospitalized with medication and observation. Additionally, the unit of day for hospital stay was relatively too large considering that our average postoperative stay was 3.28 days for surgeon A's group and 4.48 days for surgeon B's group.

Surgical recurrence is an essential indicator for surgical success in large studies^10,19^. The operation time is a sensitive indicator for the evolution of surgical proficiency^20–22^. Interestingly, operation time is an early indicator, while surgical recurrence is a later indicator for the learning curve. Surgical proficiency was achieved in 32 patients by operation time and 33 by surgical success in pelvic reconstructive surgery without mesh by surgeon A. Pelvic reconstructive surgery with mesh by surgeon B was steady after 37 by operation time and 47 by surgical success.

The strength of our study is that we retrospectively analyzed two similar large cohorts of POP by the individual surgeon. To our knowledge, we are the first study using CUSUM to evaluate surgical proficiency in vaginal pelvic reconstructive surgeries. In addition, we considered two parameters, surgical recurrence and operation time, to approach the specific procedures necessary for maturation.

Not taking into account the common parameters of surgical complications is a limitation of our study. There are two reasons we were unable to factor it into our analysis. First, some complications, such as mesh erosions, may need a more extended follow-up period to be revealed. An adequate period of follow-up for long-term or rare complications is hard to define. Second, we gathered many more in-hospital than out-of-hospital complications. The medical records might have had missing values in the retrospective cohort. Logically, the CUSUM learning curve analysis of in-hospital complication rates would find fewer procedures required than the analysis of surgical recurrence found and more procedures than operation time did. The second limitation is the learning curves can vary by individual surgeon. The third limitation is that hysterectomy volumes fell in Taiwan during our follow-up period^23^. This caused a significant difference in the rates of previous and concurrent hysterectomy in surgeon A's cohort. Because surgeon A's cohort usually included an optional vaginal hysterectomy, this result may have negatively impacted the CUSUM analysis of operation time.

We are the first study using CUSUM to analyze vaginal pelvic reconstructive surgery with or without mesh, comprising SSLF and anterior and posterior colporrhaphy by the individual surgeon. Currently, two approaches to pelvic reconstructive surgeries, abdominal and vaginal, are mainly utilized. Recent studies have focused on minimally invasive procedures through the abdominal route. The robotic sacrocolpopexy calculated proficiency in approximately 78 cases based on intraoperative complications^6^. The laparoscopic pectopexy calculated proficiency by CUSUM based on the operation time, blood loss, and length of hospital stay^7^. Nevertheless, few studies have calculated the learning curve of the vaginal route. De Tayrac analyzed the learning curve of bilateral anterior sacrospinous ligament suspension associated with anterior mesh repair but was based on the number of adverse events, which may not be as sensitive as CUSUM^20^. Although we observed that surgical proficiency based on surgical failure and operation time was achieved after fewer vaginal procedures in our series, the calculated parameters of CUSUM are different. Comparing the proficiency case numbers between vaginal and abdominal routes may need further study.

Vaginal pelvic reconstructive surgery for multiple-compartment pelvic organ prolapse is complicated. The CUSUM test was applied to find the learning curve of surgical performance when the surgical outcome, operation time, and hospitalized days reached a steady state where cumulative success rates consistently remained above the acceptable boundary line of the CUSUM analysis. Combining the results of CUSUM analysis of surgical recurrence and operation time, surgical proficiency can be achieved in 32–33 cases in reconstructive surgery (SSLF, anterior and posterior colporrhaphy) without mesh, and reconstructive surgery with mesh is steady after 33–47 patients. Our data showed that cumulative sum control chart analysis could assist in the training program of urogynecologic fellows. The trainees can visualize their performance as they progress toward surgical proficiency.

## Methods

Our study retrospectively analyzed two surgeons (Surgeons A and B) who performed multicompartment repairs of advanced pelvic organ prolapse. The two surgeons finished gynecologic training as gynecology specialists and received urogynecology training as trainees. They were at the stage of starting urogynecologic surgeries independently. We traced back to their first operation as the principal operator instead of an assistant. The data were collected at a single center longitudinally between 2004 and 2018. The study was approved by institutional review board of National Taiwan University Hospital(IRB/REC 202107084RIN). Written informed patient consents were obtained. All methods in this study were performed in accordance with the relevant guidelines and regulations. Patient medical records were retrieved, including patient age, body mass index (BMI), past medical illness, surgical history, surgical method, operation time, hospital stay, complications, and outcomes. The postoperative pain scores were recorded during the period of admission, and voiding function was evaluated. Patients in stable recovery were discharged to an outpatient department for follow-up. The minimal follow-up period was 24 months. Patients were all pelvic organ prolapse quantification (POP-Q) stage III or IV. Those who planned to undergo pelvic reconstructive surgeries were divided into two groups based on the performing surgeon (A or B). Recurrent surgeries were not included in our study.

The primary outcome measure was surgical proficiency, which was based on anatomic success. Anatomic failure was defined as the objective recurrence of POP-Q points Aa, Ba, C, Ap, Bp, or D with the Valsalva maneuver were beyond the hymen during follow-up. Surgical proficiency was defined as the point at which the CUSUM score rises above the acceptable boundary line H1 of the CUSUM analysis and remains there. Secondary outcomes were stabilization of operation time and the period of postoperative hospitalization.

The CUSUM results for anatomic success were recorded on a graph in which the x-axis represents the number of procedures, and the y-axis represents the "cumulative sum" of successes (s) and failures (1- s). With each anatomic success, the graph rises by "s"; with each anatomic failure, the graph falls by "1-s" (Supplement Table 1). When the proportion of anatomic successes to failures is sufficiently high, the CUSUM score rises above the boundary line, H1. The boundary line is set according to the acceptable failure rate, P0, and the unacceptable failure rate, P1. In our study, P0 and P1 were selected to be 10% and 20% according to the previous literature^13,24,25^. When there was no recurrence, the CUSUM graph increased by s = $P/(P+Q)$  = 0.145, where P =$\mathrm{ln}[\left(1-P0\right)/(1-P1)]$ and Q = $\mathrm{ln}[ P1/P0 ]$. When a recurrence arose, the graph fell by 1-s = 0.855. Type 1 error (α) and type 2 error (β) represent the probability of falsely defining the surgeon's performance as "acceptable" or "unacceptable," respectively. Type 1 and 2 errors of 10% were considered acceptable in this study. Proficiency was obtained when the graph crossed above H1 and remained there. It is assumed that the surgeon's performance matured with a false positive rate of α.

CUSUM was also applied to find the operation time learning curve. CUSUM analysis was used to measure the deviation between the raw data of each case and the mean value of the cohort, tracking the accumulation of each deviation in a sequential manner. Thus, CUSUM was defined as ${\sum }_{i=1}^{n}\left({\chi }_{i}-\mu \right)$, where ${\chi }_{i}$ is the operation time in each case and $\mu$ is the mean operation time of the cohort. By this method, the CUSUM curve portrays trends in data that are not discernable with other approaches^26^.

Surgeon A performed multicompartment repairs with natural tissue repair (NTR) by unilateral sacrospinous ligament fixation (SSLF) and concomitant vaginal anterior and posterior colporrhaphy. Surgeon A did not use mesh in the surgeries. Vaginal hysterectomy is an optional surgery but is usually performed. We preferred to perform SSLF with hysterectomy to have a better surgical field but preserved the uterus with mesh to avoid complications. SSLF surgery was performed with a Veronikis ligature carrier and Miya hook as previously described in detail^27–29^. Surgeon A performed anterior and posterior colporrhaphy with a traditional two-layer plication using a 2-0 Vicryl suture (Ethicon Inc, Somerville, NJ, USA).

Surgeon B preferred the same surgery with macroporous polypropylene mesh augmentation. The SSLF and anterior colporrhaphy were both augmented with the mesh. The posterior colporrhaphy was similar to that performed by Surgeon A. The surgical details are as follows. After completely separating the bladder from the vaginal wall, a purse-string suture of the posterior bladder wall excluding the bladder neck was performed using a Monocryl 2-0 suture. A polypropylene mesh (Gynemesh PS Nonabsorbable Prolene Mesh, Ethicon, US) was then trimmed to a central diamond shape with two sets of paired arms. Appropriate skin holes were created outside the obturator foramen on each side. One tunneler was used to pull the arms out of the pits via the outside-in method. The apical suspension was achieved by directly suturing the mesh to the sacrospinous ligament. The mesh was adjusted to the appropriate position under the bladder, and sutures fixed the tail of the diamond body to the upper part of the anterior cervix. The right part of the mesh head was fixed to the right side of the periurethral tissue by Vicryl 2-0 sutures (Ethicon Inc, Somerville, NJ, USA). The mesh was adjusted to prevent excessive tightness, and the anterior vaginal wall was sutured in two layers using a Vicryl 1-0 suture. An inverted T-shaped incision of the posterior vaginal wall from the introitus to the rear side of the cervix was performed after hydrodissection, and the bilateral posterior vaginal wall was separated from the rectum. The rectovaginal septum and posterior vaginal wall were sutured in two layers using a Vicryl 1-0 suture.

Statistical analysis was performed using SPSS Statistics software (version 26.0; IBM, Armonk, NY) and GraphPad Prism version 9.12 (GraphPad Software Inc.). The results are presented as the mean and standard deviation for continuous data and as the number and percentage for categorical data. A normality test was performed with the Shapiro–Wilk test prior to the 25-tier group analysis. The independent t-test and one-way analysis of variance (ANOVA), Mann–Whitney U test, and Kruskal–Wallis test were used to test shifts in normally distributed and nonnormally distributed continuous values, respectively. The chi-squared test and Fisher's exact test were used for categorical data, as appropriate. All tests were considered significant at p < 0.05.

### Ethics approval

The local institutional review board (202107084RIND) was approved by the Research Ethics Committee Office of National Taiwan University Hospital (https://www.ntuh.gov.tw/RECO/Index.action).

## Supplementary Information

Supplementary Table 1.

## Data Availability

The datasets generated and analysed during the current study are not publicly available due the privacy of patients and by the restriction from rules of the Institutional Review Board. But the datasets are available from the corresponding author on reasonable request.
