# Supplementary material for: Analyzing the learning curve of vaginal pelvic reconstruction surgery with and without mesh by the cumulative summation test (CUSUM)
Source: Sci Rep. 2022 Apr 29;12:7025. doi: 10.1038/s41598-022-11039-5 (PMC9054794; doi:10.1038/s41598-022-11039-5)
Supplement: Supplementary file 1 — Supplementary Table 1. [file 41598_2022_11039_MOESM1_ESM.docx]

**Supplement table 1.** Cumulative Sum Calculations for Varying Acceptable and Unacceptable Failure Rates

| Parameters | p0 = 5%, p1=10% | P0=10%, P1=20% | P0=20%, P1=40% |
| --- | --- | --- | --- |
| Sample weight for each success, $s=P/(P+Q)$ | 0.072 | 0.145 | 0.293 |
| Sample weight for each failure, $1-s$ | 0.928 | 0.855 | 0.707 |
| $P=\ln[ \left( 1-P0 \right)/(1-P1)]$ | 0.054 | 0.118 | 0.288 |
| $Q=ln[ P1/P0 ]$ | 0.693 | 0.693 | 0.693 |
| $a=\ln[ \left( 1-\beta\right)/\alpha]$ | 2.197 | 2.197 | 2.197 |
| $b=\ln[ \left( 1-\alpha\right)/\beta]$ | 2.197 | 2.197 | 2.197 |
| Upper failure rate threshold, $H1=b/(P+Q)$ | 2.941 | 2.709 | 2.241 |
| Lower failure rate threshold, $H0=a/(P+Q)$ | -2.941 | -2.709 | -2.241 |

The values P0 = 10% and P1 = 20% were used in the main analysis

α = 0.10 = probility of type I error; β = 0.10 = probability of type 2 error; P0 = acceptable failure rate; P1 = unacceptable failure rate
